# Mapping Intravascular Ultrasound Controversies in Interventional Cardiology Practice

**DOI:** 10.1371/journal.pone.0097215

**Published:** 2014-05-09

**Authors:** David Maresca, Samantha Adams, Bruno Maresca, Antonius F. W. van der Steen

**Affiliations:** 1 Department of Biomedical Engineering, Erasmus University Medical Centre, Rotterdam, the Netherlands; 2 Tilburg Institute for Law, Technology and Society, Tilburg University, Tilburg, the Netherlands; 3 Centre de recherche pour l'étude et l'observation des conditions de vie, Paris, France; 4 Interuniversity Cardiology Institute of the Netherlands, Utrecht, the Netherlands; 5 Imaging Science and Technology, Delft University of Technology, Delft, the Netherlands; University of Maryland, College Park, United States of America

## Abstract

Intravascular ultrasound is a catheter-based imaging modality that was developed to investigate the condition of coronary arteries and assess the vulnerability of coronary atherosclerotic plaques in particular. Since its introduction in the clinic 20 years ago, use of intravascular ultrasound innovation has been relatively limited. Intravascular ultrasound remains a niche technology; its clinical practice did not vastly expand, except in Japan, where intravascular ultrasound is an appraised tool for guiding percutaneous coronary interventions. In this qualitative research study, we follow scholarship on the sociology of innovation in exploring both the current adoption practices and perspectives on the future of intravascular ultrasound. We conducted a survey of biomedical experts with experience in the technology, the practice, and the commercialization of intravascular ultrasound. The collected information enabled us to map intravascular ultrasound controversies as well as to outline the dynamics of the international network of experts that generates intravascular ultrasound innovations and uses intravascular ultrasound technologies. While the technology is praised for its capacity to measure coronary atherosclerotic plaque morphology and is steadily used in clinical research, the lack of demonstrated benefits of intravascular ultrasound guided coronary interventions emerges as the strongest factor that prevents its expansion. Furthermore, most of the controversies identified were external to intravascular ultrasound technology itself, meaning that decision making at the industrial, financial and regulatory levels are likely to determine the future of intravascular ultrasound. In light of opinions from the responding experts', a wider adoption of intravascular ultrasound as a stand-alone imaging modality seems rather uncertain, but the appeal for this technology may be renewed by improving image quality and through combination with complementary imaging modalities.

## Introduction

Tremendous advances occurred during the last 40 years in the field of medical imaging of the heart and the coronary vasculature, triggered by the increasing need to reduce acute myocardial infarctions. The intravascular imaging route led to the development of X-ray angiography in the 1960's, balloon angioplasty and related techniques in the late 1970's and early 1980's. Meanwhile, in the early 1970's, academic research programs focused on developing two-dimensional real-time ultrasound imaging of the heart, transferring in particular knowledge from underwater acoustics to medicine [1]. This noninvasive route led to echocardiography, an imaging modality acclaimed for its radiation free nature but lacking the resolution to image the coronary vasculature.

The need for a technique able to provide high resolution images of diseased coronary artery wall structures, referred to as vulnerable plaques [2] and primarily responsible for myocardial infarctions, arose in the early 1990's when false-negative coronary angiography cases became evident [3]. Intravascular ultrasound (IVUS) is a catheter-based echocardiography modality that was patented in 1972 [4] and further developed to investigate the status of the coronary artery wall. The tip of an IVUS catheter incorporates a single piezoelectric transducer (40 to 60 MHz frequency range) or a circular array of transducers (20 MHz) to generate circular cross sectional images of the arterial wall, perpendicular to the longitudinal artery axis. Single transducer IVUS images are acquired by mechanically rotating the transducer over 360 degrees, whereas in circular array IVUS the ultrasound beam is steered electronically. The resolution of IVUS images is of the order of 100 µm in the axial direction, 300 µm in the lateral direction [5], and IVUS imaging depth typically ranges from 5 to 10 mm. IVUS technology has played an important role in the standardization of balloon angioplasty and stent treatments. Before intervention, IVUS can provide the artery lumen diameter, the plaque morphology and burden [6] thanks to the delineation of the external elastic membrane, and can be used to select optimal stent dimensions. Post intervention, IVUS is also useful to control stent apposition and possible complications. In addition, IVUS technology proved to be very useful in cardiovascular research. Since plaque progression and regression can be accurately measured with IVUS, the efficacy of new cardiovascular therapies on plaque volume can be quantified. IVUS also serves as gold standard for the evaluation of new intravascular modalities. Next to the estimation of plaque burden, the most valued IVUS function is calcium detection. Unfortunately, predicting the risk for future acute cardiovascular events requires knowledge of plaque composition [7], which is not provided by conventional IVUS. Several IVUS signal processing techniques have been developed at an academic level to augment IVUS capabilities in detecting and characterizing coronary artery plaques at risk [8], [9] but failed to reach clinical practice so far.

Looking back, the realization of IVUS is undoubtedly a technical success. Twenty years after its introduction in the clinic in the early 1990's, IVUS technology continues to bring scientific insight into the pathophysiology of the coronary artery disease and helps guiding percutaneous coronary interventions. To date, the noninvasive imaging techniques capable of identifying coronary artery wall lesions are magnetic resonance imaging (MRI) and multi-slice computed tomography (MSCT), but their resolution remains inferior to *in situ* catheter techniques. Minimally invasive imaging techniques include coronary angiography, angioscopy, IVUS, intravascular optical coherence tomography (OCT), the combination of near infrared reflectance spectroscopy (NIRS) with IVUS [10]. OCT in particular has emerged as a rival for IVUS by generating more superficial but higher resolution images of the arterial wall.

Surprisingly, IVUS innovation appears relatively limited since its introduction in the clinic, especially in terms of image quality. Significant academic innovations such as IVUS flow [11], [12], IVUS palpography [8], [13], harmonic IVUS [14], [15] and contrast-enhanced IVUS [9], [16] were to date not taken up by industry. Furthermore, IVUS remains a niche technology, whose clinical practice did not vastly expand nor disappear. IVUS reimbursement varies considerably worldwide which reveals a contrasted adoption of IVUS. In Japan, IVUS is reimbursed separately since 1994, even for diagnostic use. In the United States, IVUS is not reimbursed but procedure codes leave enough room for IVUS utilization where necessary. In the rest of the world, there is no separate reimbursement for IVUS. IVUS market penetration worldwide follows accordingly.

Following scholarship in sociology of innovations, we were interested in understanding both the reasons for current adoption practices and prospects for further adoption or development of the technology in the future. As part of this, we sought to outline issues related to the technology, which are often referred to as ‘controversies’ [17], meaning that they can still be disputed, negotiated, etc. and practice, whereby the end result is still unknown. Understanding the various issues at stake for the respondents is important because how these further develop in practice can shape the future of the technology. To identify these issues, we took a qualitative approach. This approach combined a survey of experts currently generating innovations and/or using IVUS technologies with a social network analysis of their interactions.

## Materials and Methods

To outline the dynamics of the network of experts that generates IVUS innovations and uses IVUS technologies, we conducted a survey of biomedical experts with experience in the technology and practice of IVUS. To that end, we selected a deliberative sample of potential respondents: a representative group of 49 experts dealing with the question of IVUS innovation or adjacent fields. Potential respondents were identified through publications in the field and confirmed through an expert check (Professor van der Steen, head of the Biomedical Engineering Department of the Thorax Centre, Erasmus University Medical Center, the Netherlands). Identified experts comprised interventional cardiologists, academic and corporate engineers, corporate leaders and public and private funders. For ethical considerations, the questionnaire data were collected under the agreement that data sourcing was kept anonymous. Questionnaire answers were pooled, randomized and analyzed anonymously. Participants were aware that their responses would be used in this study and that their company names may be included.

### Survey design

We developed a questionnaire [18] with a combination of open and closed questions on IVUS innovation and refined it through face-to-face interviews with three experts. After revision, the questionnaire was issued to all other experts. The questionnaire started with two open questions about coronary atherosclerosis diagnostic in humans: “*What is the best method available to diagnose human coronary atherosclerosis?*” and “*What would be an ideal technique to diagnose coronary arteries?*”. The first question permitted to review the coronary diagnostic tools that are currently appraised. The second question aimed at highlighting the limitations of existing diagnostic tools and identifying future diagnostic solutions with strong potential in the respondents' opinion. The questionnaire continued with questions focused on IVUS. To characterize the homogeneity of the respondents, we asked them to rate (from 0 - not so much, to 10 - extensive) their technical, clinical and market knowledge representation of IVUS. We collected their opinions on the advantages and disadvantages of IVUS. Next came two central questions: the room IVUS technology has left for improvement (to be rated from 0 - no room, to 10 - lots of room), and the likelihood of the existence of IVUS 20 years from now (to be rated from 0 – uncertain, to 10 - certain). These questions were inserted to quantify the future perspective of IVUS technology in the respondents' opinion. Next, we asked what the reimbursement procedure was for new medical devices in the respondent's country of residence before to specifically address the status of IVUS reimbursement. Then, we asked what were the prevailing factors that could explain the continuous but limited clinical utilization of IVUS in the respondents' opinion. Interventional cardiologists were specifically asked how IVUS helps them complete the regular tasks of their job. All these questions were inserted to collect material explaining the current adoption of IVUS. The last part contained of questions on the additional factors likely to impact IVUS innovation (e.g. educational efforts in IVUS, role of patents, and competition between experts in the IVUS market). Finally, we provided room for further comments related to IVUS technology that the respondents might want to share.

### Network analysis

**Figure 1:**
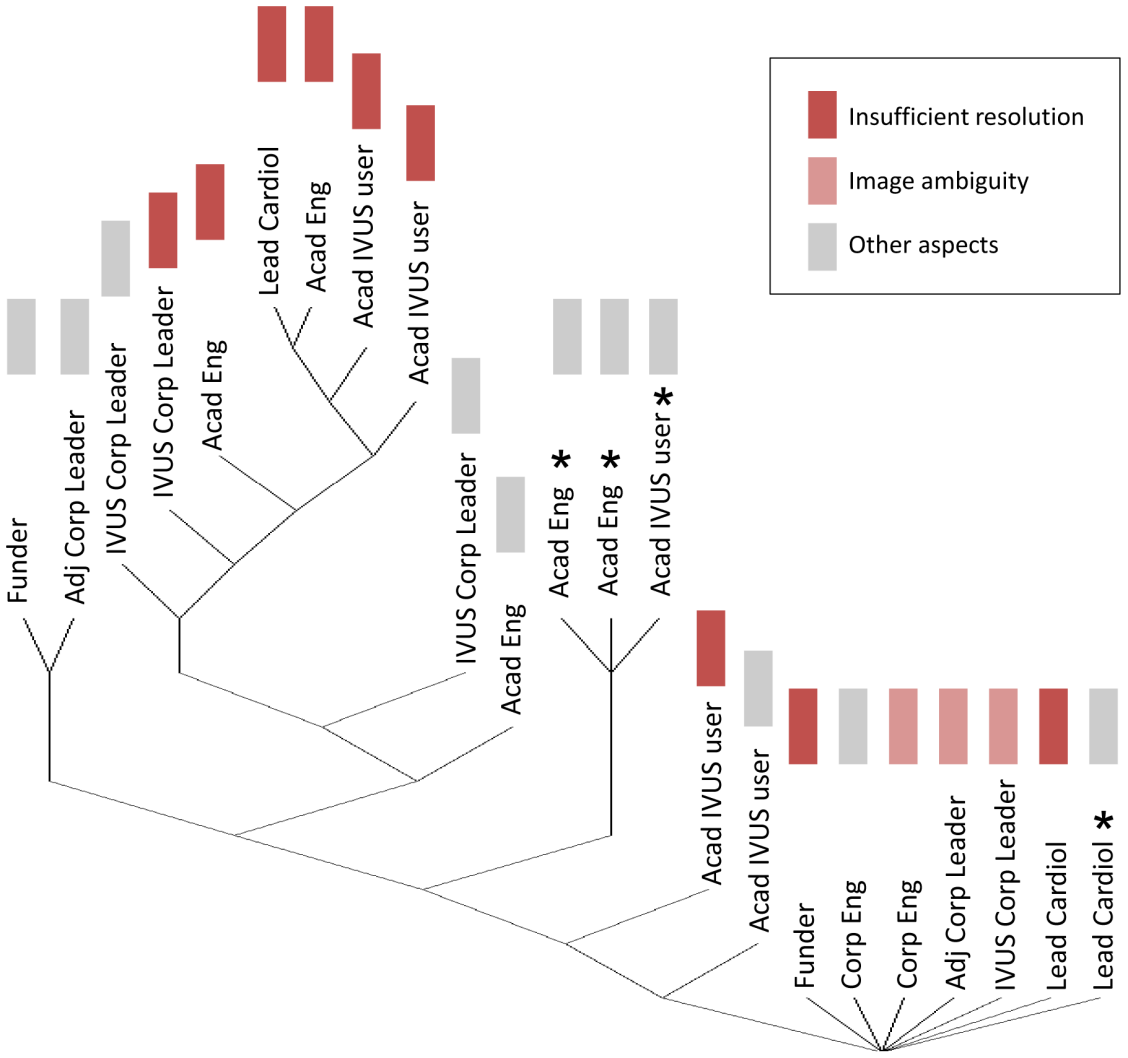
Respondents' perception of intravascular ultrasound resolution. Early experts are indicated with a star. Experts that were the least central in the network, who declared a limited level of interaction with other members, appear at the bottom of the diagram. The diagram can be subdivided as follows: a base of peripheral experts that are the least central in the network, a middle group, including early IVUS experts, with an intermediate centrality level, and finally the leading group of the network gathering the most central experts.

In the second part of the questionnaire, we asked the respondents to indicate the frequency and nature of their interaction with other identified experts. We analyzed the level of interaction among our deliberative sample of respondents using the social network analysis software UCINET (Harvard University, Boston, USA) [19]. A clique analysis was performed, assembling groups of four members or more who declared symmetric interactions [20]. Subsequently, we proceeded to a hierarchical cluster analysis of the respondents' adjacency in the network: an algorithm ordered the respondents hierarchically based on their level of similarity (amount of clique memberships shared by pairs of experts) and their proximity in the network. We displayed the result as a hierarchical clustering tree diagram using UCINET (see Figure 1). Having registered the bonds between experts, which informs on the professional network architecture, as well as the respondents' opinions on IVUS technology, we could map IVUS controversies and discuss their implications for the future of IVUS with respect to the position of the respondents in the network. It is important to realize that the individuals who contributed to this study represent a part of a bigger professional network, which is a limitation of this study. However, we postulate that the group of respondents that was surveyed is representative of the hierarchies and opinions present in the community of experts that generates IVUS innovations and uses IVUS technologies.

### Description of the respondents

With the initial list of potential respondents, 38 international institutions were represented (20 American, 9 Dutch, 3 Canadian, 2 Japanese, 2 French, 1 German, and 1 South Korean). The list comprised the following types of respondents:

- Fifteen corporate leaders, encompassing IVUS companies (Boston Scientific, Volcano, Terumo, Infraredx, Silicon Valley Medical Instruments and Colibri Technologies), a company fostering competing intravascular technologies, two general medical ultrasound companies, and a clinical research organization with experience in interventional cardiology.

- Fourteen cardiologists conducting clinical research involving IVUS or practicing IVUS-guided percutaneous coronary interventions (PCI). The group identified comprised key opinion leaders as well as international cardiologists in activity performing IVUS related research.

- Ten engineers involved with IVUS innovation or related fields. This included two academic engineers who filed constitutive IVUS inventions, four academic engineers currently in activity as well as four corporate engineers.

- Ten funders involved with IVUS innovation or related fields. Six were public funders and four private funders. Public funders represented public research organizations, public technology foundations. Private funders encompassed private technology foundations and investment firms.

Of the initial 49 experts, 23 returned a completed questionnaire. We analyzed the responses by first reassigning them into pertinent categories. Among cardiologists (8 respondents), a distinction was made between opinion-leading cardiologists (3 respondents) and academic IVUS users (5 respondents), based on internal knowledge of the field of IVUS and top authors tracking on a biomedical experts platform. Among corporate leaders (6 respondents), a distinction was made between IVUS companies (4 respondents) and related field companies (2 respondents). Public and Private funders were merged in a single group because of the limited contributions (2 respondents). Finally, engineers (7 respondents) were divided into academic (5 respondents) or corporate engineers (2 respondents).

For figure and citation purposes, we labeled IVUS corporate leaders as *IVUS Corp Leader*, corporate leaders in adjacent fields as *Adj Corp Leader*, opinion leading cardiologists as *Lead Cardiol*, academic IVUS users as *Acad IVUS Users*, academic engineers as *Acad Eng*, corporate engineers as *Corp Eng* and funders as *Funder*.

## Results

### Self-characterization of the respondents

Overall, respondents demonstrated a homogeneously high technical knowledge (total average of 8.1) and clinical knowledge (total average of 7.6) of IVUS, indicating that we successfully surveyed experts involved at the technical and medical interface of IVUS. The technical knowledge was well aligned among all categories of experts. Engineers reported a deficit in clinical knowledge (average of 6.0), below cardiologists and corporate leaders, indicating that they do not perceive themselves as medical specialists. Respondents' market knowledge appeared to be more widespread (total average of 7.0), above average for opinion-leading cardiologists and corporate leaders and below average for academic IVUS users. The experts' knowledge representation of IVUS reimbursement policies worldwide was the lowest (total average of 5.0). IVUS corporate leaders and corporate leaders of related fields were above average while corporate engineers and funders were below. Cardiologists and academic engineers were average. Corporate leaders naturally appeared to be more focused than the rest on the non-technical factors that governing the development of medical technologies.

### Historical context of IVUS introduction

Twenty years elapsed between the registration of the first IVUS patent in 1972 [4] and its transfer to clinical research in the early 1990's [21], [22], when the technology caught the attention of interventional cardiologists willing to visualize coronary artery wall lesions. This was clear, for example, in this response from a European respondent:

“As the big worry for cardiac echography was to see through the ribs, the idea of a phased array catheter was suggested. But in the meantime, the external linear array proved to be successful. People could see the heart and babies. Since the noninvasive approach worked, people forgot about the phased array catheter until the early nineties, when the need for a high resolution technique able to characterize coronary artery lesions emerged.” *(Acad Eng)*

The introduction of IVUS as a high tech medical device followed a classical path. It first started as an academic engineering project aiming at developing intracardiac echocardiography in real time. IVUS technology eventually found light as a high resolution tool to characterize coronary artery lesions, as a result of the academic collaboration of cardiologists and engineers. This was clear, for example, in this response about the advantages of IVUS from Canadian respondent:

“It [IVUS] has good penetration through blood and soft tissue, enabling estimation of vessel dimension, vessel remodeling, and plaque burden with high sensitivity and specificity in identifying coronary calcifications”. *(Acad Eng)*

The second phase of IVUS introduction was its adoption by industry. The industrial development and aim given to IVUS was largely shaped by Boston Scientific. This is evident, for example, in this response from the US:

“In the first 10 years, Mansfield/Boston Scientific and CVIS were alone; then BSC bought CVIS and merged their platform.” *(IVUS Corp Leader)*

As Boston scientific is primarily a stent manufacturer, IVUS holds an adjacent technology position within the company portfolio. IVUS was positioned as a percutaneous coronary intervention (PCI) guidance tool, allowing for peri-interventional planning and assessment of complications. This role is clear, for example, in the response of an interventional cardiologist who explains how IVUS helps him complete the regular tasks of his job:

“IVUS-guided PCI:

1. PRE: Plaque assessment, luminal diameters, stent length sizing.
2. INTRA: Stent apposition, re-entry in dissected planes, ante/retrograde chronic total occlusion (CTO).
3. POST: thrombus, edge prolapse, dissection, etc.” *(Lead Cardiol)*

This tells us that the use of IVUS evolved from a research diagnostic tool to a PCI intervention guidance tool. It raises the question of the role for intracoronary imaging technologies. To date, IVUS technology is perceived as well aligned within the product portfolio of IVUS companies. This was reported in this response from the US:

“All companies try to create a coronary artery imaging platform. For Boston Scientific and Terumo, IVUS is an adjacent market. For Volcano, Infraredx, it is a central market.” *(Funder)*

The third stage of the introduction of IVUS is its reimbursement via public health policy. Overall, the reimbursement process for a new medical device consists in the following: evidence-based medicine must prove benefits in using the technology. Subsequently, randomized clinical trials are to be conducted to determine whether the technology leads to an improved effectiveness in terms of patient outcome as well as a superior cost-effectiveness than the standard of care. The reality of IVUS reimbursement appears contrasted. In the US, the situation was reported as follows:

“There is no separate reimbursement for IVUS and it must be bundled into the existing Diagnosis-related group (DRG) for the specific coronary intervention. A separate set of cost-effectiveness and clinical utility data would be required to create stand-alone IVUS reimbursement” *(IVUS Corp Leader)*

This indicates that IVUS only partially fulfills the usual requirements for the reimbursement of a new device in the United States. Most notably, it appears that the technology has failed to demonstrate clinical utility. Yet, several respondents pointed at the clear dissymmetry in the reimbursement of IVUS that exists worldwide. This was clear, for example, in this response from the US:

“Separate reimbursement exists in Japan, where IVUS penetration is widely viewed as the deepest in any part of the world. This is not circumstantial. The second highest major market penetration is in the US, where it is not reimbursed separately but in which specific procedure codes do leave enough room for IVUS utilization where necessary. The lowest penetration exists in EU and Asia/Latin America markets where per-procedure economics are tightest and no separate reimbursement exists. Thus, while there is undoubtedly strong clinical belief in the utility of IVUS, there is an undeniable correlation between where that clinical belief manifests and where the economic policies are most accommodating.” *(IVUS Corp Leader)*

Japan's separate reimbursement of IVUS is unique worldwide and seems to be the result of a stronger belief in the utility of IVUS interventional cardiology practice. Nonetheless, the status of IVUS reimbursement is a strong indicator of a contrasted acceptance of the technology and reveals that IVUS must be engaged into a set of controversies.

### Open debates surrounding IVUS technology

By reviewing the contributors' answers, we identified six controversies revolving around IVUS technology; a controversy being defined as a debate surrounding a technique, for which the outcome has not yet been determined [17], [23]. The controversies identified are reported in Table 1.

**Table 1:** Ongoing IVUS controversies conveyed by the respondents.

Controversy	Positive responses	Negative responses
***Invasiveness of IVUS***	“Minimally invasive”; “The technique is invasive but I am an interventional cardiologist. IVUS takes 30 seconds”	“IVUS is very invasive to find the site of interest”; “A major disadvantage is that IVUS is invasive”
***Resolution of IVUS***	“High resolution and similarity to pathology”; “It has good penetration through blood and soft tissue, enabling estimation of vessel dimension, vessel remodeling, and plaque burden with high sensitivity and specificity in identifying coronary calcifications”	“Unacceptably poor resolution”; “Resolution is not enough for some particular purpose (Thin Cap Fibroatheroma)”
***Usefulness of IVUS as diagnostic tool***	“In order to understand the local problem, a catheter is the best”; “Large investigation range in combination with pull-back”; “Relatively quick, you can see obstruction, size and shape of the lesion (morphology)”; “Well validated quantitative measurements, many related outcome evidence by IVUS measurements (example, minimum stent area to predict future revascularization), easy to learn/use”; “Resolves ambiguous anatomy on angiogram, especially at left main”	“Intra-coronary imaging is too invasive and too late to use”; “most information not needed in daily practice unless complication”; “Lack of clarity of the images (I think I know what I'm looking at but not entirely sure) and the difficulty of acquiring those images”; “A catheter does not provide a complete view of the vascular tree”
***Usefulness of IVUS as PCI guidance tool***	“Inadequacy of angiography to guide clinical decision making in complex anatomy”; “Clinical trials have shown that the use of IVUS is reasonable during PCI for several indications. The medical literature continues to demonstrate limitation of angiographic-only guidance for PCI”	“Clinical impact on decision making is limited”; “There is no large clinical trial to show the benefit of using IVUS”; “Absence of evidence based medicine guidelines/Competition with FFR&OCT”
***Impact of IVUS reimbursement on IVUS innovation***	“Separate reimbursement exists in Japan, where IVUS penetration is widely viewed as the deepest in any part of the world. This is not circumstantial”; “Japan has reimbursement even for diagnostic IVUS. If not, the usage will decrease to half of now”	“Reimbursable for appropriate use”; “I think the biggest limit is the lack of investment in academic research”; “It affects the clinical use. Institutions like the Thoraxcenter simply supply the difference, but in peripheral hospitals the clinical use is affected. The IVUS innovation is an academic/industrial process and is financed by other means”
***Educational efforts in IVUS***	“A focused educational program is needed for realizing the potential of this technique”	“Today it's a niche technology, teaching efforts questionable given poor penetration”

- The first controversy concerns the invasive nature of IVUS. It opposes experts who perceive invasiveness as a limitation, e.g. by preventing the screening of asymptomatic patients, to experts who relativize the minimal invasiveness of IVUS in light of the interventional nature of their job.

- The second controversy identified concerns the resolution of IVUS imaging. It opposes experts who perceive IVUS resolution as insufficient to detect important features of atherosclerotic plaques (e.g. thrombi, thin cap fibroatheroma, plaque composition) and/or consider IVUS images as difficult to interpret, to experts who praise the sufficient clarity of IVUS whose resolution provides well validated quantitative measurements of atherosclerotic plaques (e.g. size and shape of coronary lesions, residual lumen, calcium detection, clear images of stent struts).

- The third controversy concerns the practicability of IVUS as a diagnostic tool. It opposes experts who consider that IVUS is an expensive and late diagnostic solution (restricted to patients who already need an intervention) which is tedious to analyze, to experts who praise the local knowledge of the plaque provided by IVUS and therefore its prognostic value, as well as the fact that IVUS is relatively quick to use.

- The fourth controversy concerns the utility of IVUS as a percutaneous coronary intervention (PCI) guidance tool. It opposes experts considering that IVUS has a limited impact on clinical decision making and failed to prove clinical benefits in terms of PCI treatment outcome, to experts who consider that IVUS improves safety overall by resolving ambiguous anatomy on angiograms (for example at the left main coronary artery), permits the adequate selection of stent landing zones and stent size, and allows for the evaluation of post-intervention complications.

- The fifth controversy concerns the impact of IVUS reimbursement on IVUS utilization worldwide. It opposes experts who consider that IVUS current reimbursement leaves enough room for an appropriate use and that expansion is primarily limited by the lack of demonstrated IVUS benefits, to experts who consider that a separate reimbursement of IVUS, even for diagnostic use, would favor its development as observed in Japan.

- The sixth controversy concerned the means invested in the education of IVUS experts. In light of the absence of clearly demonstrated clinical benefits in using IVUS, some experts consider that enough educational efforts are consented (live cases at conferences, publications, experts visits), while others consider that because IVUS technology is not exploited at its full potential, a global educational effort is required (e.g. exposure of cardiologists in training, creation of a certification program, online consulting systems).

It is interesting to note that only one of the six controversies identified - the ongoing debate on IVUS resolution - was intrinsic to the technology itself. All others appeared as peripheral debates surrounding IVUS practice and questioning aspects of interventional cardiology practice in general. Nonetheless, the amount of controversies identified is not negligible and raises the question of the future of IVUS, especially since the field of intracoronary imaging has become more competitive with the introduction of OCT. In particular, the role of intravascular imaging seems to be questioned: is the goal to mimic histology, to perform prognostic imaging and/or to provide procedure guidance?

### Mapping IVUS controversies

In order to analyze controversies by taking into account the social network dynamics, we projected the respondents' opinions on the UCINET tree diagram (Methods section). In Figure 1, we projected the opinions of the respondents who mentioned “resolution” when answering to the question of the disadvantages of IVUS. By doing so, we mapped the ongoing debate on the resolution of IVUS imaging (identified earlier as the only one intrinsic to the technology of IVUS). It appeared that the six most central experts in the network perceive IVUS resolution as insufficient. Interestingly, the figure reveals that none of the historic experts (marked with a star in the figure) cited resolution as a disadvantage of IVUS. From their perspective, IVUS was introduced as a high resolution technique, filling a void in interventional cardiology. Figure 1 displays therefore the evolution of the debate on resolution since the introduction of IVUS. We know that IVUS image quality did not drastically change since its introduction. Still it changed from high to low at the advent of OCT. A clear change in understanding of what “high resolution” means is observed, independent from the developmental path of IVUS technology: it indicates a mutual shaping between technology and society.

### Future perspective for IVUS technology

**Figure 2:**
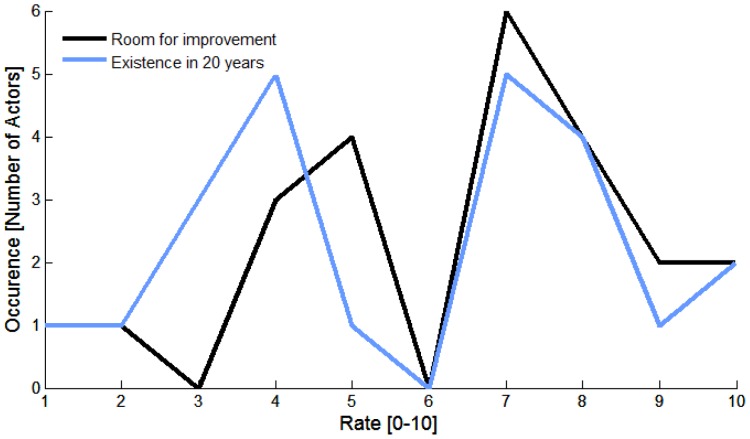
Future perspective of intravascular ultrasound according to the respondents.

When looking at the distribution of the answers of the respondents to the two central questions (the room for improvement left for IVUS technology and the perspective of existence in 20 years), we observed a singular double-peak distribution (Figure 2).

**Figure 3:**
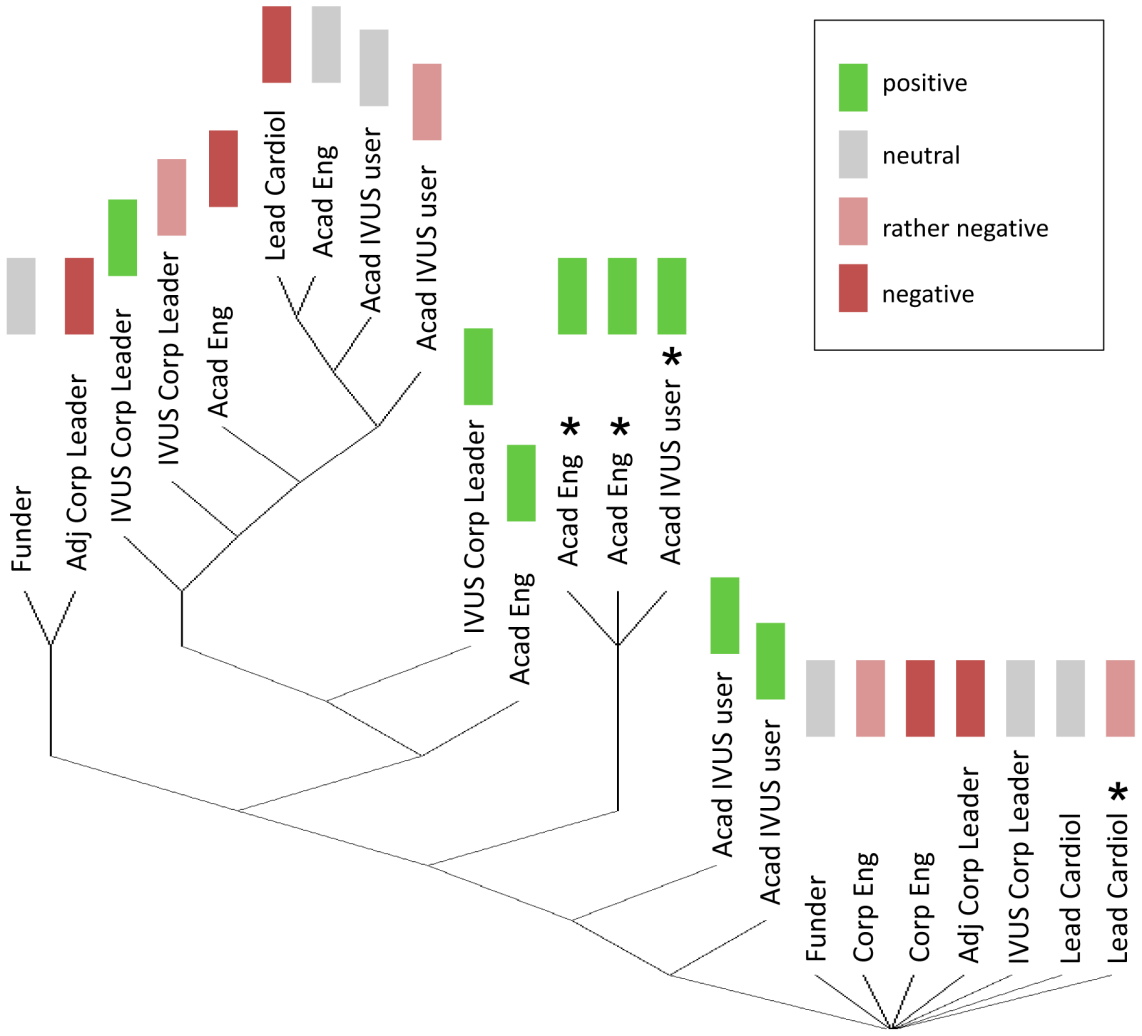
Room for innovation in intravascular ultrasound according to the respondents. Early experts are indicated with a star. Least central experts in the network appear at the bottom of the diagram and most central experts at the top.

This result clearly indicates that two populations coexist in the IVUS network surveyed, one optimistic and one pessimistic about the technology perspective. We rearranged the responses into two categories, the positive opinions (values above 6) and the negative opinions (values below 6). Academic engineers were the most optimistic when rating the room for improvement that IVUS technology has (average of 7.8), which is natural considering that they are professionally invested in IVUS innovation. Academic IVUS users were optimistic as well (7.3) followed by IVUS corporate leaders (6.8). On the contrary, corporate engineers appeared pessimistic (4.5) while other corporate leaders were the most pessimistic (1.5). Logically, it appears that the judgment on the room for improvement of IVUS is directly dependent on the degree of investment the experts have in IVUS: engineers and corporate leaders involved with other intravascular technologies were negative about the innovation potential that IVUS has left. Results were projected on the tree diagram in Figure 3, showing that historic experts emerge as the principal subgroup sharing a positive opinion about the room for innovation left for IVUS technology. Note that most central experts, who are currently professional active, conveyed a rather negative opinion.

Concerning the existence of IVUS in 20 years, the optimistic group gathered academic IVUS users (7.1), academic engineers (6.6) and IVUS corporate leaders (6.5). The pessimistic group was made of the opinion-leading cardiologists (4.3), other corporate leaders (4.0) and funders (3.3). Engineers and IVUS corporate leaders were convinced of the future of the technology. Conversely, opinion-leading cardiologists and funders were skeptical about the future of IVUS. Again, historic experts shared an optimistic vision of the future of IVUS as for the previous question. Central experts in the network appeared more balanced. Experts with a low centrality (potentially less tied to IVUS) were rather pessimistic.

When the answers to both questions are coupled, it appears that academic engineers, academic IVUS users and IVUS corporate leaders share a globally optimistic view, whereas opinion-leading cardiologists, other corporate leaders and funders shared a globally pessimistic view. Therefore, a major conclusion is that opinion-leading cardiologists and funders, who orientate innovation due to their position, disclosed a pessimistic opinion of the existence of IVUS technology 20 years from now. Their regard on the future of the technology was clearly exposed, for example, in this response from the US:

“In my opinion, the use of IVUS only makes sense as part of conventional angioplasty. If it were incorporated as part of the procedure, then there would not be a need for a second invasive procedure. However, the fact that IVUS is invasive may limit its capability for growth, especially if non-invasive MRI and CT coronary diagnostic imaging capabilities reach the point that they become more attractive, competitive diagnostic procedures. This may be possible in the near future, and might decrease the need for invasive diagnostic procedures like IVUS.

A more important consideration would be the results of comparative effectiveness studies of IVUS, compared to conventional angioplasty. If it could be proved that IVUS increased longevity or decreased complications, this would contribute toward making this procedure more attractive to practitioners” *(Funder)*

## Discussion

This study demonstrates that the development and positioning of IVUS, in the high-tech environment of interventional cardiology adjunctive devices, is shaped by co-developing controversies surrounding the technology. We questioned why IVUS neither expanded nor disappeared since its introduction 40 years ago. The capacity of IVUS to estimate total coronary plaque burden was almost unanimously reported by the respondents and appears as the principal advantage of IVUS. IVUS is the only clinical tool capable of measuring plaque burden in vivo [24] and was shown to be a predictor of major adverse cardiovascular events [6]. For this reason, the technology is praised in cardiovascular research since it allows for longitudinal studies of atherosclerosis progression. IVUS can also rely on an extensive publication database and has become the gold standard to compare to when introducing a new intravascular imaging technology. Therefore, one possible explanation is that it is the utilization of IVUS in cardiovascular research that kept the technology running in academic medical centers.

On the other hand, the absence of good reimbursement was clearly reported as directly limiting the clinical use of IVUS (or would affect its use in the case of Japan). But respondents from the United States, Europe and Japan alike also reported that the creation of stand-alone reimbursement for a medical device requires cost-effectiveness and clinical utility data. Therefore, we can pose the question whether it is the lack of demonstrated medical evidence in favor of IVUS technology that prevented the creation of a stand-alone IVUS reimbursement, with the exception of Japan where the technology was reimbursed in 1994.

The case of IVUS reimbursement in Japan can be explained by two converging factors. First, an early interest for IVUS technology (in 1993 IVUS was used in Japanese hospitals for clinical research), which was originally offering both the highest resolution available in interventional cardiology and validated quantitative and outcome related measurements (e.g. “minimum stent area to predict future vascularization”). Second, a reimbursement accreditation procedure for approving a new medical device that is “not inferior to existing alternatives but does not have to be superior in every aspect”. Other countries adopted a wait-and-see stance, as a stand-alone IVUS reimbursement requires a set of “cost-effectiveness and clinical utility data”. Penetration of IVUS is the lowest in Europe, Latin America and Asia where reimbursement policies are the least accommodating. It is the combination of a strong belief in the clinical utility of IVUS and an accommodating economic policy that fosters the use of the technology. A Japanese respondent reported that if IVUS was not reimbursed in Japan, its use would be halved compared to now. Ulucanlar et al. [25] recently argued that ‘technology identities’ e.g. their novelty, effectiveness, utility, risks and requirements are socially constructed and shape technology adoption. Here we show in the case in the particular case of IVUS that the ‘identity’ of a given technology can vary geographically.

Several other lessons relevant for both the future of IVUS and the introduction of new intracoronary imaging techniques can be learned from this sociological study of IVUS innovation. We observed that the only controversy intrinsic to IVUS technology was the debate on IVUS resolution. It is important to analyze at the architecture of the network of IVUS experts surveyed when evaluating the implications of this controversy. The tree diagram (Figure 1) can be subdivided in three groups: a base of peripheral experts that are the least central in the network, a middle group of early IVUS experts with an intermediate centrality level and finally, the leading group of the network gathering the most central experts. Experts at the top of the diagram are likely to be in close collaboration and/or competition with each other and to be the most deeply involved with IVUS or adjacent technologies. Furthermore, their point of view is dominant because of their central position in the network. But they are also likely to have the highest interest in promoting a given imaging modality. Since the six most central experts in this survey are all indicating that IVUS resolution is insufficient, we can assume that improvements are mandatory for the future of the technology. Note that a significant enhancement in IVUS image quality could be achieved by improving IVUS lateral resolution, currently three times worse than axial resolution [5]. This strategy was investigated academically by Chandrana et al. [15] but did not materialize industrially to date.

Interestingly, we observed that the middle group of early experts who were involved with the introduction of IVUS pointed at other IVUS limitations than resolution. Certainly because, from their perspective, IVUS filled a void in the interventional cardiology space and entered as the highest resolution modality. On the contrary, peripheral experts are likely to be involved to a lesser extent with IVUS and potentially give a more positive judgment of the advantages and disadvantages of IVUS technology. This observation is in line with the concept of “certainty trough” developed by Donald A. MacKenzie [26], which states that knowledge producers from a peripheral discipline attribute less uncertainty to technology from another discipline than those involved directly in knowledge production. More than resolution, it is the ambiguity of IVUS images that the respondents incriminate, telling us that understanding IVUS images requires expertise. Whether a higher resolution will solve image interpretation issues is not clear. Despite its microscopic resolution, OCT is still in a phase of standardization, proven benefits have not been demonstrated yet and the technology is not exempt of artifacts, which makes OCT image interpretation an expert's task as well.

Several respondents stressed the need for focused educational programs in order to fully realize the potential of intracoronary imaging techniques. They criticized the lack of exposure of medical doctors to these techniques at resident stage. Educational efforts in IVUS were considered as “hobbyist” by an early European practitioner. And the relevance of educational efforts was questioned by others given the poor penetration of IVUS. When characterizing themselves, engineers declared a deficit in clinical knowledge of IVUS compared to other groups, whereas technical knowledge was homogeneously high among experts. A more extensive education of engineers to the reality of IVUS clinical procedures and catheter laboratory workflow might prove to be critical for the successful introduction of future intracoronary imaging modalities. Complementarily, educating interventional cardiologists further in the physics of intravascular imaging could improve patient treatment, by helping them to recognize image artifacts and hence secure their diagnosis. It is likely that a more substantial knowledge overlap between interventional cardiologists, biomedical engineers, industry leaders and investors would accelerate IVUS innovation. The evolution of cardiac interventions based on new medical technology was shown to be progressing along co-evolving pathways: advances in scientific understanding indeed, but more important improvements of the ability to develop and use medical technologies as well as learning in medical practice [27].

Most central experts in the network had a skeptical perception of the potential for innovation that IVUS technology has left (Figure 3). This can be understood from a historical perspective as the field of IVUS faced a relative lack of IVUS innovation in the past 20 year. 40 MHz IVUS was reported as early as 1991 in scientific literature [20], [21] and remains the standard product of major IVUS companies today; image quality in IVUS did not experience major breakthroughs in 20 years. Several respondents criticized the lack of competition in the IVUS market, which potentially stifles innovation. These respondents also called for the creation of new start-ups to re-energize clinical translation. IVUS companies happened to sit on innovation in some cases therefore limiting the dissemination of new technological developments. It was reported that patents have played a role, but that most of them are now obsolete. Academic engineers were the only ones that appeared clearly positive about the potential left for IVUS innovation (Figure 2), probably because they envision potential refinements in IVUS technology.

Given that five of the six identified controversies are external to IVUS technology, we can hypothesize that decision making at the industrial, financial and regulatory levels will orientate predominantly future innovations in intracoronary imaging. As reported earlier, a major conclusion is that opinion-leading cardiologists and funders, who shape innovation to a large extent, disclosed a pessimistic opinion of the existence of IVUS technology 20 years from now. In general, minimally invasive imaging modalities are logically not perceived as adequate for the early screening of coronary atherosclerosis in asymptomatic patients. First, because the information they provide is local (as opposed to a full view of the coronary anatomy e.g. angiography/MRI/CT) and second because of their invasive character. Therefore, wide adoption of intravascular imaging will depend on added benefits for cardiac patients requiring intervention. If a medical consensus advocates the identification of flow limiting lesions, then the combination of fractional flow reserve (FFR) and angiography is sufficient [28]. If medical guidelines state that the assessment of plaque vulnerability is critical, then intravascular imaging techniques will play a role as prognostic tools. In any case, next generation IVUS technologies as well as new intravascular technologies will compete with OCT in terms of image quality and guidance of stent apposition, and with the combination of angiography and FFR in terms of clinical decision making.

## Conclusion

To date, IVUS remains valuable as it is the only clinical tool capable of imaging plaque burden *in vivo* and because it is grounded in extensive scientific literature. Mapping IVUS controversies revealed that the appeal of intravascular ultrasound may be renewed by improving (lateral) image resolution and/or through combination with other imaging modalities. An integrated IVUS-OCT catheter, providing OCT resolution and IVUS penetration simultaneously, was recently evaluated *in vivo* [29]. This technical solution has the merit to solve the issue of IVUS resolution. Other combined modalities were mentioned such as NIRS-IVUS [30] and intravascular photoacoustics [31]. These may provide an enhanced characterization of the arterial wall but will still need to act on IVUS image quality. In all cases miniaturization and integration of independent modalities will weigh on cost-effectiveness.

The successful translation of future intravascular imaging technologies in interventional cardiology practice will require a rapid demonstration of clinical utility, which is a necessary condition for an efficient reimbursement; otherwise, hospitals cannot afford to use it. Finally, this must be coupled on a willingness of care practitioners to gain experience in a range of quickly developing technologies [32] in order to improve patient care. Unless, of course, in the advent of preventive medicine, the amount of percutaneous coronary interventions decreases drastically.

The future of IVUS as a stand-alone modality appears uncertain due to a lack of demonstrated benefits of the technology in terms of patient outcomes. Moreover, its use in cardiovascular research is likely to erode with the emergence of newer intravascular imaging techniques. As time passes, competition among adjunctive interventional cardiology devices increases, whereby the chance that IVUS will reach stand-alone reimbursement decreases.
